# Supplementary material for: Effect of fluid intake on cognitive function in older individuals: A prospective study
Source: PLoS One. 2025 Oct 6;20(10):e0333793. doi: 10.1371/journal.pone.0333793 (PMC12500155; doi:10.1371/journal.pone.0333793)
Supplement: Attachment — Submitted filename: Reviewers comments.doc [file pone.0333793.s001.doc]

Shigihara et al. conducted a study involving 33 Japanese elderly people. The aim of the study was to evaluate the associations between fluids intake and cognitive performances. More specifically, they assessed cerebral blood dynamics. The authors concluded that elderly people with higher fluid intake showed more improvements in their cognition, which is based on the changes in cerebral blood dynamics. Cerebral blood dynamics can be used to monitor patients’ dehydration states, which affect their cognitive status.

**Major comments.**

The sample size is only 33 individuals, and they are all from a single geriatric health service facility. The sample has a high degree of homogeneity, which may lack broad representativeness. As a result, it is difficult to extrapolate the research findings to a more general elderly population. It is recommended that in future studies, the sample size be expanded and elderly people from different regions and with diverse life backgrounds be included. Furthermore, It is impossible to assess the patients' fluid intake and dehydration status before admission, which may lead to biases in the research results. Although fluid intake was recorded during the study period, the situation before admission is unknown, which may interfere with the judgment of the relationship between fluid intake and cognition.

Followed are the details.

1.     Abstract: The characteristics of the survey subjects is not well defined. It is unknown whether they are healthy, and if they have chronic diseases. During the research process, it is unclear how many subjects withdrew, and for what reasons. Also, the average age of the subjects and the duration of the study are not provided. The authors just showed the “several months”, three months? Six months? Please clarify.

2. The study lack of Control Group: This study lacks a control group, making it difficult to determine that changes in cognitive status are solely due to fluid intake. For example, during the intervention period, factors such as changes in nursing methods and living environment may also affect cognitive status, but it is impossible to distinguish their effects from those of fluid intake in this study. It is recommended to add a control group in future research, such as setting parallel groups with different fluid intake standards, or comparing two groups of elderly people receiving regular care and enhanced fluid intake care, so as to more accurately assess the impact of fluid intake on cognitive status.
3. As for the methods measuring the cognitive performances, the MMSE-J and FAB were used to assess cognitive status, which may not fully reflect all aspects of cognitive function. Aspects such as executive function and other different dimensions of memory were not fully covered in this study. As shown in previous studies, the Short-term memory and attention are the most vulnerable to the effects of dehydration. But in this study, the aspects of the cognitive performances were not assessed. Moreover, when assessing brain activity, relying solely on several parameters measured by carotid ultrasound is difficult to comprehensively reflect the complex hemodynamic changes in the brain. Techniques such as functional magnetic resonance imaging (fMRI) and transcranial Doppler ultrasound (TCD) can be considered to obtain richer brain hemodynamic data.
4. Statistical Analysis: The characteristics of the elderly people was not displayed in this part, such as the gender, age, height, weight, and if they were healthy or had some chronic diseases. I recommend the authors to add the information. When dealing with multivariate relationships, using only Pearson correlation analysis and bootstrapping methods is somewhat insufficient. In the case of multiple confounding factors such as age and underlying diseases, multiple linear regression analysis should be considered to clarify the independent contributions of various factors to changes in cognitive status. Data Normality Test: The article does not mention conducting normality tests on the data, and some statistical methods (such as Pearson correlation analysis) have certain requirements for data distribution. If the data do not meet the normality assumption, it may lead to biases in statistical results. It is recommended to supplement data normality tests and select appropriate statistical methods according to the data distribution, such as non - parametric tests.
5. Results Section: In the "Results" section, the description of some key results is not detailed enough. For example, when referring to the improvement of participants' cognitive status, it only states that "More than one - third of the participants showed an increase in MMSE-J scores after the study period", without specifying the specific proportion and number of people, which is not conducive to readers' accurate understanding of the results. Specific proportion and number information should be supplemented to enhance the persuasiveness of the results. When describing the relationship between ultrasonic parameters and cognitive status, only correlation coefficients and P - values are listed, lacking an explanation of the practical significance. For example, when stating that MMSE-J2 is negatively correlated with lPI2, lRI2, and rRI2, it should be further explained what this negative correlation means clinically and how it actually affects the cognitive function of the elderly.
6. References: The number of references in this article is too small, and there are only seven references from the past five years (7/34). It is recommended that the author conduct a new literature review.

This article needs to be revised. I do not recommend its publication.
