# Supplementary material for: Effect of fluid intake on cognitive function in older individuals: A prospective study
Source: PLoS One. 2025 Oct 6;20(10):e0333793. doi: 10.1371/journal.pone.0333793 (PMC12500155; doi:10.1371/journal.pone.0333793)
Supplement: Attachment — Submitted filename: Response-to-Reviewers.docx [file pone.0333793.s002.docx]

**Response to reviewers:**

To the editor and reviewers:

Thank you for the opportunity to submit a revised version of our manuscript entitled ‘Effect of fluid intake on cognitive function in older individuals: A prospective study’.

We appreciate the time and effort you have invested in reviewing our manuscript. Your insightful comments and constructive feedback have been invaluable in enhancing the quality and clarity of our study. We have carefully revised the manuscript to address all of your suggestions and concerns. We displayed all changes in blue font instead of using the Track Changes function in MS Word, as there were too many edits in the 'Revised Manuscript with Track Changes.docx' file.

Please find below our point-by-point responses to each of the comments.

**Response to Reviewer 1 Comments**

**Comment 1-1.**

Abstract

The abstract is lengthy but is lacking in key aspects. Primarily of concern, there is no actual data given for results. This is of absolute importance and the key statistical analyses and numerically reported outcomes must be presented.

**Response 1-1:**

Thank you for your insightful comment. We have revised the Abstract to include specific figures, such as *P*-values to enhance clarity and precision. We hope the revised abstract addresses the reviewer’s concern satisfactorily.

**Comment 1-2.**

Introduction

The second and third sentence begin with the word "It". Be more direct in your writing style.

**Response 1-2:**

Thank you for your valuable suggestion. In accordance with the suggestion, we have revised the sentence (lines 43–45 in the revised manuscript). In addition, the manuscript has been edited by the same professional English editing service as in the previous version. We hope the revised text now reads more naturally.

**Comment 1-3.**

Line 62: Should be no y after thirst.

**Response 1-3:**

Thank you for your suggestion. We have removed the letter ‘y’ in the revised manuscript (line 53 in the revised manuscript).

**Comment 1-4.**

You need to develop a proper problem statement.

**Response 1-4:**

Thank you for your valuable feedback. This comment made us realise that the original introduction lacked clarity and consideration. In response, we re-organised the latter half of the introduction and added a new Fig 1 to accurately convey the study concept and hypothesis. Additionally, we revised the entire Discussion section to ensure consistency with the updated introduction. We appreciate the opportunity to improve the manuscript.

**Comment 1-5.**

Line 66: Your hypothesis statement occurs before your purpose statement.

**Response 1-5:**

Thank you for your valuable comment. In accordance with your suggestion, we have completely re-organised the latter half of the Introduction section. We hope that the revised version meets the reviewer’s expectations.

**Comment 1-6.**

Methods
My main concern is the design of the study.

It is never explicitly stated how fluid intake was measured. For example, did patients drink out of metered containers and the change in mass or volume was assessed?

**Response 1-6:**

Thank you for your valuable comment. We apologize for not explicitly highlighting this aspect in the previous manuscript, despite its central importance to the study. In response to your feedback, we have revised the manuscript in lines 137 to 142 to address this point.

As a clinical recommendation within the facility, all older residents were advised to maintain a fluid intake of approximately 1,500 mL per day. Staff members, including nurses, therapists, and care workers provided fluids such as water or green tea in cups marked with a scale, both at the bedside and in the refectories during meals and upon request. The amount of fluid consumed was documented in the residents’ clinical records as a part of routine clinical practice. We have referenced these clinical records in our study.

**Comment 1-7.**

You have a within subjects design, but there seems to be no true control and intervention arm. First assessment was taken between 0-16 days after admission. Then, a second assessment occurred approximately 83 days later. The induction of blinded, reduced or increased fluid intake after a few weeks of hospitalization would have been a much stronger design. The ultrasonographic score changes with reduced or increased FI could have been confirmed, and then you could even have simply tested cognition during periods of lower and higher cererbral blood flow dynamics parameters.

**Response 1-7:**

Thank you for your insightful comment regarding our study design. We apologize for the insufficient description in the previous version. In accordance with your suggestion, we added a new Fig 1 that illustrates the study concept and protocol at a glance. We hope this addition adequately addresses your concerns.

One of the primary objectives of the current study is to examine the relationship between fluid intake and changes in cognitive state (Theme 1 in Fig 1). The analyses are based on correlation analyses and do not require a control group (lines 240–241 in the revised manuscript). Although a more robust design might involve randomized induction of fluid intake levels, such an approach would be ethically problematic, as increased fluid intake is recommended by the EFSA guidelines as part of standard clinical practice [1]. We aim to encourage participants to consume approximately 1,500 mL of fluid per day, aligning with clinical recommendations. Providing instructions to limit or alter fluid intake without clinical indication would be inappropriate and unethical. However, we remain vigilant about the potential adverse effects of excessive fluid intake, particularly concerning the risk of heart failure (details provided in the revised Introduction and the left panel of Fig 1B). This concern motivated the design of our study. Although we acknowledge that a randomized controlled approach might be preferable from a purely scientific perspective, we believe that the current study design is the most ‘practical’ and ethically acceptable within the clinical setting.

**Comment 1-8.**

No background or explanation of the cognitive status questionnaires is given.

**Response 1-8:**

Thank you for your valuable comment. In accordance with your suggestion, we have added the relevant description in lines 166–179 in the revised manuscript. The MMSE is the most widely used tool for dementia screening [2], primarily assessing learning and memory performance [3]. The FAB is another neuropsychological assessment designed to evaluate frontal lobe function concisely [4], an area in which the MMSE is less sensitive [5]. Both MMSE-J and FAB are scored on scales of 0–30 and 0–18, respectively; lower scores indicate more severe cognitive impairment. These two assessments were selected for the following reasons. First, the MMSE-J is routinely used in our facility as a part of standard clinical practice. Second, the clinical staff are familiar with the FAB, which is less time-consuming and more sensitive to certain cognitive domains that the MMSE may not effectively evaluate [6]. We hope that the revised manuscript satisfactorily addresses the reviewer's concerns.

**Comment 1-9.**

Number of participants: There are so many missing pieces of data it is difficult to follow the paper. All participants with missing data need to be removed. The abstract makes it appear that the n is almost double of the participants applicable in one of your most important figure (i.e. Figure 1) when the 3 outliers were removed. I am also not convinced the rationale for removing this data is valid.

**Response 1-9:**

Thank you for your helpful comment. In accordance with your suggestion, we have re-organised the entire manuscript—from the introduction to the discussion. We acknowledge that the original manuscript’s description was somewhat complex, which may have obscured the core objectives. Your feedback highlighted that the study's purpose was not clearly articulated and that essential and trivial information were intertwined. In the revised version, we summarised the main topic into two ‘Themes’, which are visually summarised in the new Fig 1. Simply put, the study was motivated by a clinical question: Should we encourage older individuals to increase fluid intake for better cognition without restrictions? (lines 70–73 in the revised manuscript) Clinicians often hesitate to promote increased fluid intake due to concerns about the risk of heart failure [lines 64–70 in the revised manuscript and the new Fig 1B]. Based on this, we hypothesized that excessive fluid intake might not enhance cognition, drawing an analogy from the relationship between blood volume and cardiac output. It is intuitively understandable that very high fluid intake does not improve MMSE-J scores, as demonstrated by the reversed U-shaped curve in the new Fig 2A (lines 410–413 in the revised manuscript).

In the revised manuscript, all analyses are categorized into three groups:

Theme 1: Comparison between fluid intake and changes in cognitive state

Theme 2A: Comparison between fluid intake and ultrasonographic parameters

Theme 2B: Comparison between cognitive state and ultrasonographic parameters

Theme 2B consists of three sub-comparisons (lines 255–256 in the revised manuscript).

Theme 2b-1: Comparisons at the first assessment

Theme 2B-2: Comparisons at the second assessment

Theme 2B-3: Comparisons of changes between the first and second assessments

(See in the revised Methods)

We eliminated analyses that were not central to our primary objectives.

These five analyses (Theme 1, 2A, 2B-1, 2B-2, and 2B-3) are essentially independent, and mismatches in the number of data points do not influence each other (lines XX–XX in the revised manuscript). Although it is possible to adjust all analyses to the dataset with the smallest number of data points, doing so would result in the loss of valuable information obtainable from the entire dataset. Thus, we chose to utilize the full available dataset, despite some missing data, except for the PI variable. We excluded PI from the revised manuscript due to significant missing data and because it does not provide additional important insights beyond those gained from the other analyses.

We hope we have correctly interpreted the underlying message behind comments 1–9 and have addressed it appropriately in the revised manuscript.

**Comment 1-10.**

No validation for the instrument used for determining BFP is provided. Did you see high variance in the patients? I may have overlooked where this outcome's data are located, but if no, why not just base relative fluid intake off body mass?

**Response 1-10:**

Thank you for clarifying regarding the LBM. In accordance with your suggestion, we have added the relevant description in lines 143–147. Distribution of bodily fluids varies across different body components: approximately three-fourths of the total body water is distributed within lean body mass, making it a more appropriate measure for evaluating body size [7–9]. The body fat percentage values were 27.9±8.2% (range: 10.9%–44.8%) at the first assessment and 31.0±7.6%(range: 9.8%–42.1%) at the second. We used a household-use body fat scale, categorised as a health meter. In Japan, the accuracy of such health matters is regulated by the Ministry of Economy, Trade and Industry. Additionally, all the body fat percentages were measured using the same device throughout the study to maintain consistency. Any potential measurement errors should be mitigated during the statistical analyses, even if the device has some inherent inaccuracies.

**Comment 1-11.**

There is just an overload of tables and figures. It is difficult to follow.

**Response 1-11:**

Thank you for pointing this out. We completely agree with the reviewer’s perspective and have re-organised the entire manuscript, from the Introduction section to the Discussion section.

We removed unnecessary analyses, figures, and tables, and focused on the data that support the main objectives of the study. The core concept of the study is now accurately illustrated in the revised Fig. 1 and is categorized into two themes. We hope that the revised manuscript effectively conveys our key messages.

**Response to Reviewer 2 Comments**

**Comment 2-1.**

Major comments.

The sample size is only 33 individuals, and they are all from a single geriatric health service facility. The sample has a high degree of homogeneity, which may lack broad representativeness. As a result, it is difficult to extrapolate the research findings to a more general elderly population. It is recommended that in future studies, the sample size be expanded and elderly people from different regions and with diverse life backgrounds be included.

Furthermore, It is impossible to assess the patients' fluid intake and dehydration status before admission, which may lead to biases in the research results. Although fluid intake was recorded during the study period, the situation before admission is unknown, which may interfere with the judgment of the relationship between fluid intake and cognition.

**Response 2-1:**

Thank you for your valuable comment. In accordance with your suggestion, we have revised the manuscript accordingly. With regard to the first point about the ‘homogeneity’ of study participants, we agree with the reviewer and have added this as a limitation in the manuscript (lines 504–507). Specifically, the critical amount of fluid intake is likely to vary among different groups of older individuals. If the present study is published, we plan to expand it by collaborating with other facilities within our hospital network and outside of our group, involving a larger number of participants to obtain more robust and generalizable results.

With regard to the amount of fluid intake prior to admission, we cannot entirely exclude its potential influence on the present results. However, we assume that the severity of dehydration prior to admission was randomly distributed across all participants, and our statistical analyses should have mitigated its effect. Although the uncontrolled levels of dehydration before admission may reduce the sensitivity of our analyses and increase the risk of type II error, we still observed significant correlations between mnFI and changes in cognition and other factors. Thus, believe this limitation is unlikely to substantially impact our main conclusions. We have added this explanation to the Limitations section (lines 507–515 in the revised manuscript).

**Comment 2-2.**

Abstract: The characteristics of the survey subjects is not well defined. It is unknown whether they are healthy, and if they have chronic diseases. During the research process, it is unclear how many subjects withdrew, and for what reasons. Also, the average age of the subjects and the duration of the study are not provided. The authors just showed the “several months”, three months? Six months? Please clarify.

**Response 2-2:**

Thank you for your helpful comment. Although we have already described some participant profiles, such as age, sex, and diagnosis, in Tables 1 and 2, we acknowledge that this information was not clearly organized and lacked a distinction between essential and non-essential information details. In the revised manuscript, we present a more concise and structured description of participant characteristics—such as age, sex, weight, lean body mass, and comorbidities—in the main manuscript (lines 96–101 and 276–283) as well as in Table 1. According to the study period (between the first and second assessments), it was 82.6 ± 14.9 days (from 34 to 119 days). Although this was mentioned in the previous version, it was not sufficiently prominent. In the revised manuscript, we have added a new Fig 1 illustrating the study concept and protocol, explicitly including the assessment interval (82.6 ± 14.9 days). Due to clinical limitations and constraints, the duration between assessments varied across participants. To maintain clarity, we have kept the expression ‘several months’ in several places throughout the manuscript.

A total of 33 participants were enrolled in the study, and none of them withdrew from the study. However, some data are missing due to clinical constraints—such as some participants not completing the second assessment by the data collection deadline (23 May 2023) or variations in participants’ mood on assessment days. We emphasize that none of the participants withdrew from the study (line 201 in the revised manuscript). Throughout the process, we prioritized participants’ dignity and autonomy and accepted the presence of missing data.

**Comment 2-3.**

The study lack of Control Group: This study lacks a control group, making it difficult to determine that changes in cognitive status are solely due to fluid intake. For example, during the intervention period, factors such as changes in nursing methods and living environment may also affect cognitive status, but it is impossible to distinguish their effects from those of fluid intake in this study. It is recommended to add a control group in future research, such as setting parallel groups with different fluid intake standards, or comparing two groups of elderly people receiving regular care and enhanced fluid intake care, so as to more accurately assess the impact of fluid intake on cognitive status.

**Response 2-3:**

Thank you for your helpful comment. We agree that employing a control group and conducting two group comparisons would be ideal in future studies; however, we are uncertain whether this approach would be ethically acceptable in the current context.

Instead, we adopted an alternative approach that is ethically safer: correlation analyses between three factors: fluid intake, cognitive state, and ultrasonographic parameters. In this study, we did not influence any clinical practices, including the amount of fluid intake. As an observation study, it involves minimal ethical concerns. This approach eliminates the need for a control group group as all analyses are based on correlational relationships (from 240 to 241 in the revised manuscript).

As regards the causes of cognitive improvement, we agree that nursing care and other factors (i.e. living environment) likely influence changes in cognition. The amount of fluid intake is only one component among these factors. We are discussing the ‘additional influence’ of fluid intake on other variables, which we have addressed with the best efforts possible (lines 236–240 in the revised manuscript). Although standard nursing care and medical support were consistently provided, improvement is still possible, particularly regarding the optimisation of fluid intake to achieve better outcomes. This rationale underpins our focus on fluid intake in the present study. We hope the response satisfactorily addresses the reviewer’s concerns.

**Comment 2-4.**

As for the methods measuring the cognitive performances, the MMSE-J and FAB were used to assess cognitive status, which may not fully reflect all aspects of cognitive function. Aspects such as executive function and other different dimensions of memory were not fully covered in this study. As shown in previous studies, the Short-term memory and attention are the most vulnerable to the effects of dehydration. But in this study, the aspects of the cognitive performances were not assessed. Moreover, when assessing brain activity, relying solely on several parameters measured by carotid ultrasound is difficult to comprehensively reflect the complex hemodynamic changes in the brain. Techniques such as functional magnetic resonance imaging (fMRI) and transcranial Doppler ultrasound (TCD) can be considered to obtain richer brain hemodynamic data.

**Response 2-4:**

Thank you for your suggestion. In accordance with your comment, we added relevant descriptions in lines 173 to 179 and lines 187 to 189 in the revised manuscript.

We agree with the reviewers’ comments that using more optimised neuropsychological assessments and neuroimaging techniques would be ideal. However, due to the limitations inherent in conducting the study within a geriatric health service facility, we had to make a compromise.

In terms of neuropsychological assessments, we selected the MMSE and FAB for two reasons (lines 173–179 in the revised version). First, the MMSE-J is routinely used in the facility as part of clinical practice. However, it is less sensitive to some important cognitive domains, such as frontal functions [5]. To address this, we included the FAB, which clinical staff are familiar with and can administer efficiently. Second, assessments needed to be concise and less time-consuming as therapists performed them amid their busy clinical duties. Although the MMSE was already integrated into routine practice, asking therapists to administer additional assessments was challenging. The FAB was the only feasible option accepted by them.

Concerning neuroimaging, we acknowledge that other techniques such as fMRI and TDC could be more suitable for this type of study. Our expertise lies in magnetoencephalography. We have published a similar study using magnetoencephalography [10], conducted in a hospital setting rather than a geriatric health service facility. Although using magnetoencephalography, fMRI, or TDC would be ideal, we chose ultrasonography for the following reasons (lines 187–189 in the revised version). First, we do not have access to TDC. Second, transferring participants from the facility to our imaging centre—located just 1 km away and equipped with fMRI and magnetoencephalography—was impractical. All measurements had to be conducted within the facility, making ultrasonography the most feasible option. However, we believe that it is acceptable to reference our previous study, which demonstrated associations between ultrasonographic parameters and magnetoencephalographic parameters sensitive to brain activity [10]. In future studies, once this study is published, we plan to utilize MEG and fMRI to further explore these relationships.

**Comment 2-5.**

4.Statistical Analysis: The characteristics of the elderly people was not displayed in this part, such as the gender, age, height, weight, and if they were healthy or had some chronic diseases. I recommend the authors to add the information.

When dealing with multivariate relationships, using only Pearson correlation analysis and bootstrapping methods is somewhat insufficient. In the case of multiple confounding factors such as age and underlying diseases, multiple linear regression analysis should be considered to clarify the independent contributions of various factors to changes in cognitive status. Data Normality Test: The article does not mention conducting normality tests on the data, and some statistical methods (such as Pearson correlation analysis) have certain requirements for data distribution. If the data do not meet the normality assumption, it may lead to biases in statistical results. It is recommended to supplement data normality tests and select appropriate statistical methods according to the data distribution, such as non - parametric tests.

**Response 2-5:**

Thank you for your helpful suggestion. In accordance with your comment, we revised the manuscript from 98 to 101, lines from 276 to 283, and lines from 224 to 233.

We added information about ‘patients’ diseases’ in the main manuscript (lines 98–101) and included ‘bodily profiles (weights and LBM)’ (lines 276–283 in the revised manuscript), although these details were previously described in Tables 1 and 2. We did not include participants’ sex and heights in the main manuscript as we consider them non-essential for the present study; however, they can be found in Table 1.

In terms of multiple linear regression analysis, we acknowledge the potential influence of confounding factors and agree that applying such analysis would be ideal if a larger dataset were available. However, our total sample size is only 33 with fewer than 20 participants included in the main analysis for Theme 1 (Fig 1). Patients have various underlying diseases, which makes them difficult to handle as confounding factors. As described below, the research field exploring the relationship between fluid intake and healthy cognition is still emerging, with limited scientific evidence. We believe more published data are necessary before delving into detailed confounding factors. After establishing an initial overview of the relationship (Fig 1), future studies should consider the influence of potential confounders.

Concerning the assumption of normal distribution, we agree with the reviewer’s point. Given our small sample size, it is impractical to assume normality. Therefore, we replaced Pearson’s correlation coefficient with Spearman’s rank correlation, coupled with non-parametric bootstrapping. Thank you for your valuable advice (lines 224–233).

We hope our explanation satisfies the reviewer.

**Comment 2-6.**

5.Results Section: In the "Results" section, the description of some key results is not detailed enough. For example, when referring to the improvement of participants' cognitive status, it only states that "More than one - third of the participants showed an increase in MMSE-J scores after the study period", without specifying the specific proportion and number of people, which is not conducive to readers' accurate understanding of the results. Specific proportion and number information should be supplemented to enhance the persuasiveness of the results. When describing the relationship between ultrasonic parameters and cognitive status, only correlation coefficients and P - values are listed, lacking an explanation of the practical significance. For example, when stating that MMSE-J2 is negatively correlated with lPI2, lRI2, and rRI2, it should be further explained what this negative correlation means clinically and how it actually affects the cognitive function of the elderly.

**Response 2-6:**

Thank you for your helpful suggestions. In accordance with the reviewer’s comment, we completely re-organised the Results section. In the previous version, we simplified the descriptions to avoid complex expressions. In the revised version, we included as many detailed numerical results as possible and removed non-essential analyses to achieve the goal of the present study. Furthermore, we added summaries at the end of each subsection to highlight the practical significance of the findings. We hope that the revised adequately addresses the reviewer’s concerns and improves the clarity and comprehensiveness of the Results section.

**Comment 2-7.**

6.References: The number of references in this article is too small, and there are only seven references from the past five years (7/34). It is recommended that the author conduct a new literature review.

This article needs to be revised. I do not recommend its publication.

**Response 2-7:**

Thank you for carefully reviewing the details of our manuscript. We agree that the number of references is limited and that many are relatively old. As highlighted in a recent article [11], the importance of avoiding dehydration for maintaining healthy cognition has been overlooked, and only a limited number of studies have addressed this topic (lines from 378 to 389 in the revised manuscript). As clinicians, we are cautious about encouraging older individuals to increase fluid intake due to the potential risk of heart failure, which can be caused by excessive fluid intake [12–14], despite the EFSA guideline recommending increased fluid intake [1]. We recognize that more published evidence is needed to support such recommendations and to better understand the potential mechanisms linking increased FI and cognitive improvement. The lack of sufficient scientific studies motivated us to conduct this study. We hope that our study will contribute to the development of this emerging field and stimulate further research, ultimately leading to increased publications. This progress can help us provide the best treatment and care for older individuals.

References

1.	EFSA Panel on Dietetic Products Nutrition and Allergic (NDA) Scientific Opinion on Dietary reference values for water/European Food Safety Authority. EFSA J. 2010;8.

2.	Shigemori K, Ohgi S, Okuyama E, Shimura T, Schneider E. The factorial structure of the mini mental state examination (MMSE) in Japanese dementia patients. BMC Geriatr. 2010;10:36.

3.	Dinomais M, Celle S, Duval GT, Roche F, Henni S, Bartha R, et al. Anatomic correlation of the mini-mental state examination: a voxel-based morphometric study in older adults. Schwarz AJ, editor. PLoS One. 2016;11:e0162889.

4.	Dubois B, Slachevsky A, Litvan I, Pillon B. The FAB: a Frontal Assessment Battery at bedside. Neurology. 2000;55:1621-1626.

5.	Bak TH, Mioshi E. A cognitive bedside assessment beyond the MMSE: the Addenbrooke’s Cognitive Examination. Pract Neurol. 2007;7:245-249.

6.	Hoshi H, Hirata Y, Kobayashi M, Sakamoto Y, Fukasawa K, Ichikawa S, et al. Distinctive effects of executive dysfunction and loss of learning/memory abilities on resting-state brain activity. Sci Rep. 2022;12:1-10.

7.	Wang Z, Deurenberg P, Wang W, Pietrobelli A, Baumgartner RN, Heymsfield SB. Hydration of fat-free body mass: new physiological modeling approach. Am J Physiol. 1999;276:E995-E1003.

8.	Sawka MN, Cheuvront SN, Carter R. Human water needs. Nutr Rev. 2005;63:S30-39.

9.	Armstrong LE. Assessing hydration status: the Elusive Gold Standard. J Am Coll Nutr. 2007;26:575S-584S.

10.	Matsumoto T, Hoshi H, Hirata Y, Ichikawa S, Fukasawa K, Gonda T, et al. The association between carotid blood flow and resting-state brain activity in patients with cerebrovascular diseases. Sci Rep. 2021;11:1-11.

11.	Nishi SK, Babio N, Paz-Graniel I, Serra-Majem L, Vioque J, Fitó M, et al. Water intake, hydration status and 2-year changes in cognitive performance: a prospective cohort study. BMC Med. 2023;21:1-17.

12.	Díez-Villanueva P, Jiménez-Méndez C, Alfonso F. Heart failure in the elderly. J Geriatr Cardiol. 2021;18:219-232.

13.	Danielsen R, Thorgeirsson G, Einarsson H, Ólafsson Ö, Aspelund T, Harris TB, et al. Prevalence of heart failure in the elderly and future projections: the AGES-Reykjavík study. Scand Cardiovasc J. 2017;51:183-189.

14.	Montalto M, D’Ignazio F, Camilli S, Di Francesco S, Fedele M, Landi F, et al. Heart failure in older patients: an update. J Clin Med. 2025;14:1982.
